# Complexes of HXeY with HX (Y, X = F, Cl, Br, I): Symmetry-Adapted Perturbation Theory Study and Anharmonic Vibrational Analysis

**DOI:** 10.3390/molecules28135148

**Published:** 2023-06-30

**Authors:** Bartosz Dzięcioł, Irina Osadchuk, Janusz Cukras, Jan Lundell

**Affiliations:** 1 Department of Chemistry, University of Warsaw, 02-089 Warsaw, Poland; 2 Department of Physics, Faculty of Science, Graduate School of Science, The University of Tokyo, Tokyo 113-8654, Japan; 3 Department of Chemistry and Biotechnology, School of Science, Tallinn University of Technology, 12618 Tallinn, Estonia; 4 Department of Chemistry, University of Jyväskylä, 40014 Jyväskylä, Finland

**Keywords:** noble-gas compounds, noble-gas complexes, xenon compounds, SAPT, intermolecular interaction energy, vibrational analysis, anharmonicity

## Abstract

A comprehensive analysis of the intermolecular interaction energy and anharmonic vibrations of 41 structures of the HXeY⋯HX (X, Y = F, Cl, Br, I) family of noble-gas-compound complexes for all possible combinations of Y and X was conducted. New structures were identified, and their interaction energies were studied by means of symmetry-adapted perturbation theory, up to second-order corrections: this provided insight into the physical nature of the interaction in the complexes. The energy components were discussed, in connection to anharmonic frequency analysis. The results show that the induction and dispersion corrections were the main driving forces of the interaction, and that their relative contributions correlated with the complexation effects seen in the vibrational stretching modes of Xe–H and H–X. Reasonably clear patterns of interaction were found for different structures. Our findings corroborate previous findings with better methods, and provide new data. These results suggest that the entire group of the studied complexes can be labelled as “naturally blueshifting”, except for the complexes with HI.

## 1. Introduction

Although noble gases were long considered inert, we now know that they may form compounds, which are interesting because of their octet-rule-breaking origin. Compared to compounds formed by other elements, these compounds are not numerous; however, the quest to find new ones has been ongoing since Neil Bartlett’s seminal discovery of the first xenon compound [1,2]. Since then, many successful syntheses, as well as theoretical and computational developments, have followed [3,4,5,6]: among them, syntheses conducted in low-temperature matrices, using a method developed in Helsinki [7,8,9,10].

The molecules synthesised in low-temperature matrices in Helsinki have the general formula HNgY, where Ng denotes a noble-gas atom, and Y is an electronegative group, e.g., F${}^{-}$, Cl${}^{-}$, OH${}^{-}$. The molecules are obtained by co-deposition of noble gas with HX precursor molecules (X = Cl, Br, I, OH, etc.). After the deposition, the material is irradiated with ultraviolet (UV) radiation, leading to photodissociation of the precursor. The subsequent migration of mobile atomic species, induced by annealing, leads to the formation of HNgY molecules. This method allowed the synthesis, for example, of the first ever compound of argon: the HArF molecule [11,12]. From quantum-mechanical simulations, it is known that the atoms in the positive (H–Ng)${}^{+}$ part are covalently bonded [4,7,9,13,14,15,16], although this can be described as a charge-shift bond [15]. The bonding between the (H–Ng)${}^{+}$ moiety and the negative Y${}^{-}$ moiety is predominantly of ionic nature.

Because these molecules are metastable—i.e., their structures do not represent global energy minima on the potential energy surface—they are very sensitive to their surroundings, which was immediately noticed in experiments and calculations. Attempts to simulate the condensed phase of HNgY molecules were unsuccessful [17,18,19], and their complexes were predicted to have short lifetimes at room temperature. Larger complexes appeared unstable, and followed a rapid reaction through a two-body (2B: HNgY → HY + Ng) or a three-body (3B: HNgY → H + Ng + Y) decomposition channel [5,18]; however, their complexation with a limited number of other molecules turned out to have a great stabilising effect on the HNgY molecule itself: this was evidenced by the fact that in the complexes, HNgY molecules typically exhibited a blueshift in the vibrational modes of $\nu_{\mathrm{HNg}}$ [20], which was attributed to the strengthening of the H–Xe bond.

The subject of noble-gas compounds and, especially, their complexes is relevant in many fields. Despite the fact that some larger complexes of HNgY are predicted not to be very stable under normal conditions, their existence is still hypothesized in low-temperature stellar environments [19], high-pressure environments relevant to the geological question of missing xenon [21,22,23], and in phase boundaries. The ArH${}^{+}$ molecular ion has been discovered in the Crab Nebula [24]. The properties of such species are important in the research of the history of interstellar matter. Molecular clouds have a temperature range of about 10–20 K, so even less stable noble-gas compounds and complexes may be relevant in this field, especially because in such environments the chemical composition is not controlled thermodynamically [25]. Moreover, the properties and chemistry of gaseous xenon are important in Earthly conditions. In addition to the missing xenon problem, xenon is known for its anaesthetic properties [26,27]. Despite many attempts to explain them, these anaesthetic properties are still mysterious, and lack support from computational studies [28,29]. If xenon penetrates cells to exert its anaesthetic action, one may suppose that it can form short-lived compounds or complexes, or even noble-gas-compound complexes in the phase-boundary-rich biochemical environment, interacting with cellular structures, and influencing the biochemical equilibria of the living cell [29].

Consequently, as the study of xenon properties has the greatest implications, its compounds have received the most attention. Xenon has the highest polarizability among noble gases: due to this fact, it was early predicted to be able to form compounds [30]. Indeed, xenon forms a large number of compounds and complexes compared to other noble-gas elements, including the HXeY family of compounds: among these, the HXeOH molecule has been studied extensively [10,14,17,18,19,31]. The different properties of other complexes have also received considerable attention, both experimentally and computationally: the reported studies include HXeCl/Br/I⋯H${}_{2}$O [32,33], HXeCl/Br⋯HCl/Br [20,34], HXeI⋯HBr/I [35], HXeI⋯HCl and HXeI⋯HCCH [36], (HXeF)${}_{2}$ and (HXeF)${}_{3}$[37], HXeF⋯HF [13,34,38], HXeBr/Cl⋯HCl/Br and HXeCCH⋯CO${}_{2}$ [14], HXeBr⋯CO${}_{2}$ [39], HXeSH⋯H${}_{2}$O/H${}_{2}$S [40], and HXeOH⋯H${}_{2}$S. Recently, Zhang et al. [15] investigated a large set of systems, i.e., HXeY⋯HX (Y = Cl/Br/I and X = OH/Cl/Br/I/CCH/CN), and established the charge-shift nature of the H-Xe bonding in the noble-gas molecule.

Although there exists a rich literature on chosen aspects of the chosen HXeY⋯HX complexes and their groups, a comprehensive analysis of the physics of the interaction, encompassing all combinations of Y and X moieties, is missing. In this study, we aimed to fill this gap, by providing a description of the nature of the interaction in the complexes of the family of HXeY compounds (Y = F, Cl, Br, I) with a series of hydrogen halide molecules HX (X = F, Cl, Br, I), using the symmetry-adapted perturbation theory [41], and combining this description with anharmonic frequency analysis. A particular focus was on the complexation effects on the Xe-H and H-X stretching modes, which were of special importance for the interpretation of the experimental spectra. In our analysis, we included the high-energy structures of complexes, i.e., the structures of local minima, because noble-gas low-temperature matrices—as opposed to, e.g., nitrogen matrices—tend to preserve the different gas-phase structures of complexes, by trapping them among noble-gas atoms, and thereby the interaction strength tends to be enhanced in the matrix [42].

## 2. Results

### 2.1. Structures

**Figure 1:**
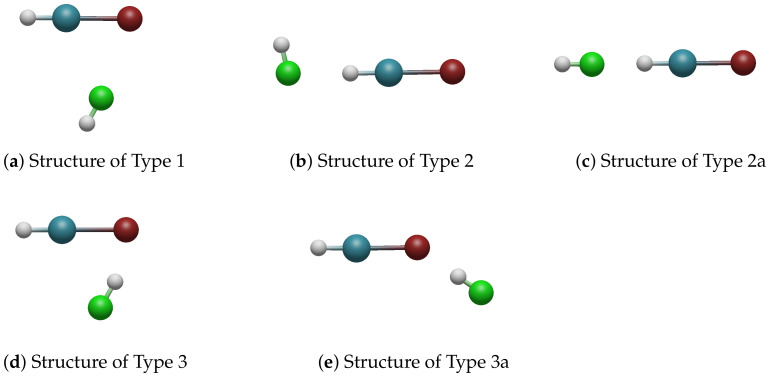
The obtained structure types of the HXeY┄HX complexes. The elements are colour-coded (H: white; Xe: teal; halogen in HX: green; halogen in HXeY: red).

Geometry optimization provided five different structure types for most of the compositions of the complexes, as shown in Figure 1. We may distinguish:

1. Type 1 structure, where the hydrogen atom of HX was directed away from the noble-gas molecule, and the halogen atoms were neighbouring each other; there was no hydrogen bond;
2. Type 2 structure, which was a bent hydrogen-bonded structure, where the hydrogen atom of the noble-gas molecule was involved in hydrogen bonding; HNgY was acting as a proton donor;
3. Type 2a structure, which was the same as Type 2, but was linear; HNgY was again the proton donor;
4. Type 3 structure, which was similar to Type 1, but the orientation of the HX molecule was reversed, with its hydrogen atom pointing toward the halogen atom of the noble-gas molecule exhibiting a hydrogen bond; HX acted as the proton donor;
5. Type 3a structure, which was similar to Type 3, but the angle formed between both interacting molecules was larger; HX was the proton donor.

Some of the investigated structures were previously reported and studied experimentally and computationally [13,14,34,36,38,40]. Zhang et al. [15] recently studied the H-Xe bonding in all these structures, except 2a and 3a, for Y and X = Cl, Br, I. Our structural results are in excellent agreement with these previous reports.

Since a given composition of the studied complexes can assume different structures, we indicate the structure of the composition by its type following the formula, i.e., HXeY⋯HX/*n*, where *n* is the structure type. For instance, HXeCl⋯HBr/2 for the complex of HXeCl molecule with HBr molecule with both molecules arranged in type 2 geometry.

### 2.2. The Total Intermolecular Interaction Energy Values

The computed values for the supermolecular interaction energy and the total SAPT interaction energy are given in Table 1. When focusing on the MP2 supermolecular energies, it can be seen that structures of Types 3 and 3a had the lowest values, ranging from ca. −20 to ca. −60 kJ mol${}^{-1}$: these could generally be considered the most stable structures. Next, in terms of stability, were the Type 1 structures that involved heavier halogen atoms in the HX unit: their interaction energies ranged from ca. −10 to ca. −14 kJ mol${}^{-1}$. Type 1 structures with light halogen atoms in HX, and Type 2 structures, had the highest interaction energy values: ca. −4 to ca. −6 kJ mol${}^{-1}$. The Type 2 structures were bound the weakest.

The data in Table 1 indicate that the total interaction energies for all Type 2 structures were very close to one another. The same can be observed for Group 3. Independent of the ingredients of the complex, and excluding complexes containing HF, the energies ranged from ca. −20 to ca. −30 kJ mol${}^{-1}$ for Type 3 structures, and from ca. −4 to ca. −6 kJ mol${}^{-1}$ for Type 2 structures. Meanwhile, the energies for Type 1 structures were very different, and clearly increased when going from HF to HI (with the exception of HXeF). The cause for this different trend seems to have been the difference in the origin of the interaction.

As depicted in Figure 1, all the structure types, except Type 1, were formed representing hydrogen bonding. For Type 3 and 3a structures, the hydrogen bond was strong, because it was formed between the strongly negative Y${}^{-}$ moiety of the noble-gas molecule and the hydrogen atom of the HX molecule, where the HX molecule acted as the proton donor. The magnitude of this interaction is comparable to the interaction in the water dimer [43,44]. The hydrogen bonds in Type 2 structures were weaker because the noble-gas molecule was the proton donor. It has been shown [14] that complexation has a shortening effect on $r_{H-\mathrm{Ng}}$, making it a less effective proton donor. Following on from the energetically unfavourable orientation, the interaction appeared weaker. However, the values of the interaction energies within the entire Group 2 were of comparable magnitude to one other, and not so diverse as for the structures in Group 1.

Conversely, the interaction origin for Type 1 structures was different. As can be seen in Figure 1a, the interaction arose from the close vicinity of the halogen atoms in both interacting molecules, and thus, the interaction energy was different for each interacting case: it systematically increased with the atomic number of the halogen atom in the HX molecule; however, for a given HX molecule, it remained remarkably constant when changing the halogen atom in the HXeY molecule. Therefore, the magnitude of the interaction energy in Type 1 structures was primarily governed by the type of HX molecule.

We now compare the energy trend within each group, separately. In the case of Group 3, the total energy slightly increased (i.e., was less stabilizing) when going from HF to HI for a given noble-gas molecule. This correlated well with the decreasing dipole moment in the sequence of HF-HCl-HBr-HI. This trend, however, was reversed in Type 1 and 2 structures. This may have been caused by the relative orientation of the dipole moments of both molecules, and is discussed in Section 2.3, in terms of SAPT contributions to the interaction energy.

In summary, the hydrogen-bonded complexes tended to have intermolecular interactions of comparable magnitude for a given geometry, independent of the composition. Conversely, the magnitude of the interaction for the complexes interacting via halogen atoms varied, depending on the halogen hydride. Simultaneously, it must be noted that complexes containing fluorine atoms are often exceptions to these considerations. The HXeF⋯HF complex was previously computationally reported by Jankowska and Sadlej [38], and by Mohajeri and Bitaab [13], in the forms of the Type 3 and 2a structures. Jankowska obtained −63.99 kJ mol${}^{-1}$ for the Type 3 structure, and −8.18 kJ mol${}^{-1}$ for the Type 2a structure, employing the MP2 method. Mohajeri and Bitaab reported −56.94 kJ mol${}^{-1}$ for the Type 3 structure, with the DFT/BMK approach. Our results agree with their data, giving the value of −62.99 kJ mol${}^{-1}$ at the MP2 theory level. The results discussed are also in agreement with previous studies of the complexes of HXeBr and HXeCl molecules [14,20].

### 2.3. The SAPT Analysis of the Intermolecular Interaction Energy

We now discuss the different terms, as obtained by the SAPT method. Aside from structures containing fluorine, all the studied complexes had a repulsive sum of first-order SAPT energies, i.e., the electrostatic and exchange interactions were not sufficient to explain the existence of the found minima, which means that they were stabilized by higher-order interaction terms: namely, by induction and dispersion.

**Figure 2:**
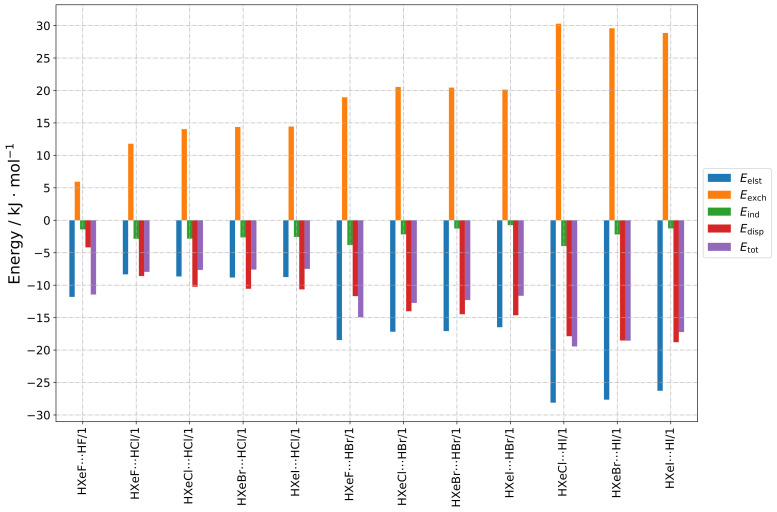
Bar graphs depicting the SAPT decomposition of the intermolecular interaction energy for HXeY⋯HX complexes assuming the Type 1 structure. The graphs are grouped by HX molecule.

**Figure 3:**
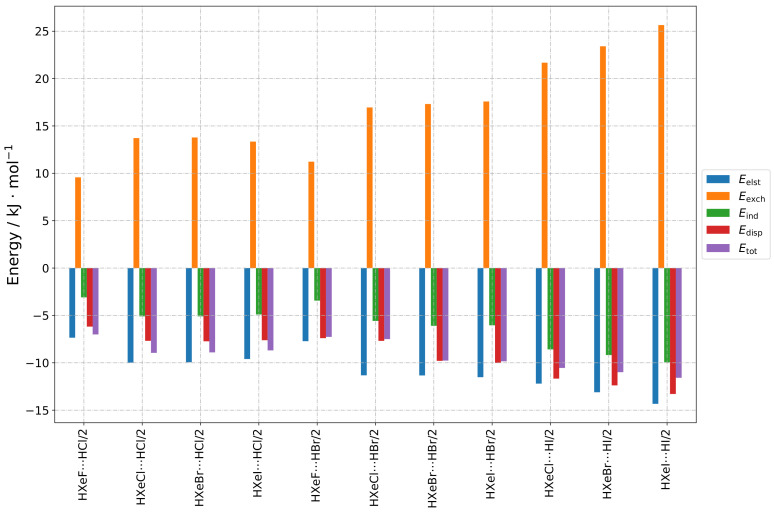
Bar graphs depicting the SAPT decomposition of the intermolecular interaction energy for HXeY⋯HX complexes assuming the Type 2 structure. The graphs are grouped by HX molecule.

**Figure 4:**
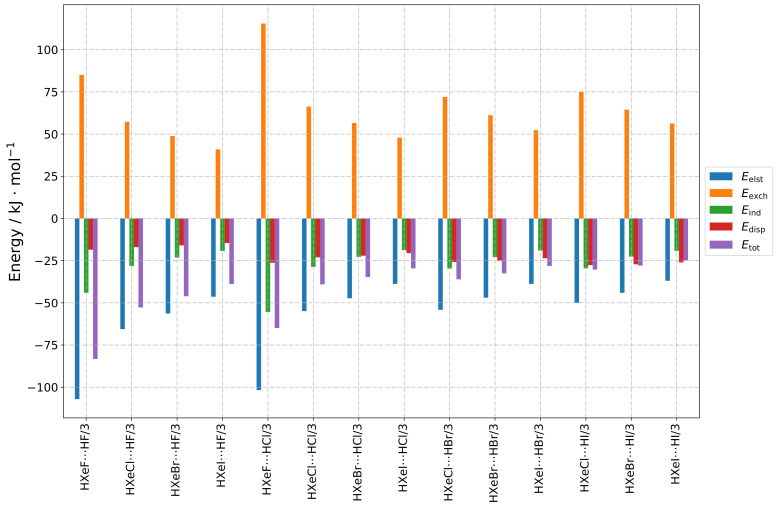
Bar graphs depicting the SAPT decomposition of the intermolecular interaction energy for HXeY⋯HX complexes assuming the Type 3 structure. The graphs are grouped by HX molecule.

**Figure 5:**
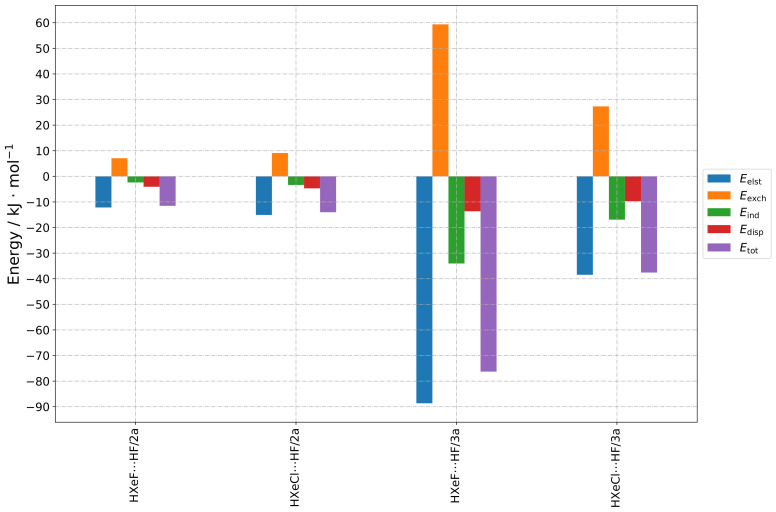
Bar graphs depicting the SAPT decomposition of the intermolecular interaction energy for HXeY⋯HX complexes assuming the Type 2a and 3a structures.

**Figure A1:**
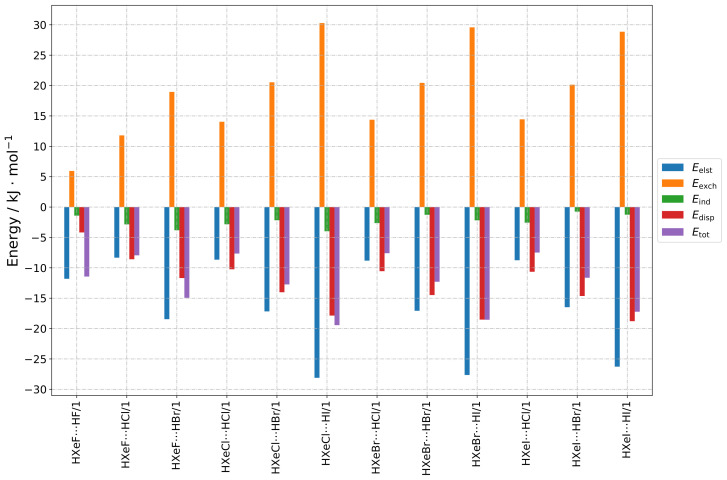
Bar graphs depicting the SAPT decomposition of the intermolecular interaction energy for HXeY⋯HX complexes assuming Type 1 structure. The graphs are grouped by HXeY molecule.

**Figure A2:**
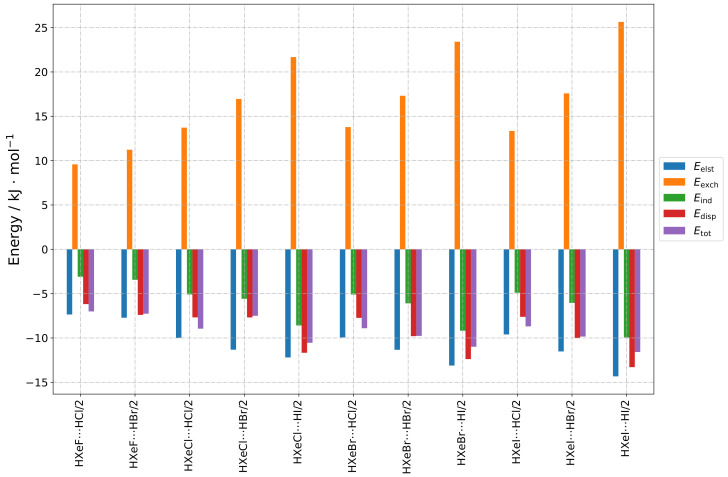
Bar graphs depicting the SAPT decomposition of the intermolecular interaction energy for HXeY⋯HX complexes assuming Type 2 structure. The graphs are grouped by HXeY molecule.

**Figure A3:**
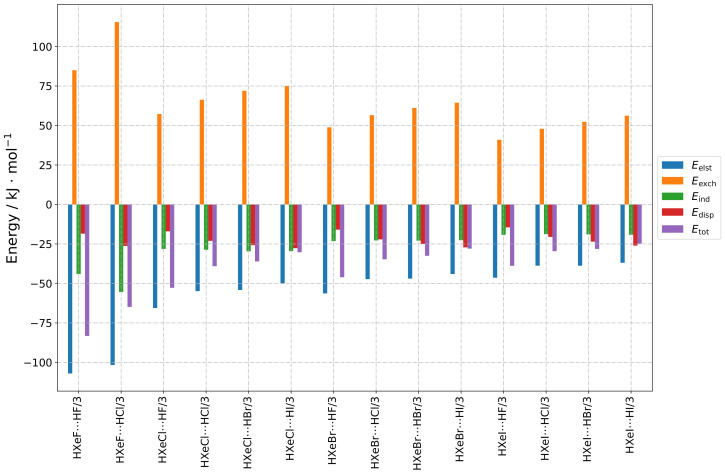
Bar graphs depicting the SAPT decomposition of the intermolecular interaction energy for HXeY⋯HX complexes assuming Type 3 structure. The graphs are grouped by HXeY molecule.

**Figure A4:**
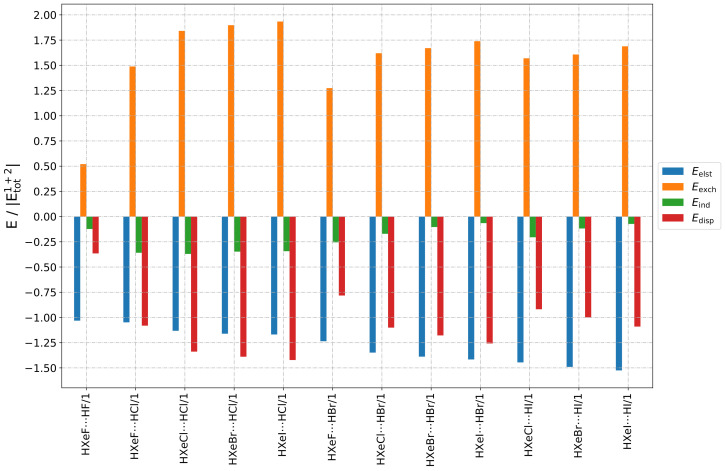
Bar graphs depicting the SAPT decomposition of the intermolecular interaction energy, using values relative to the total interaction energy, for HXeY⋯HX complexes assuming Type 1 structure. The graphs are grouped by HX molecule.

**Figure A5:**
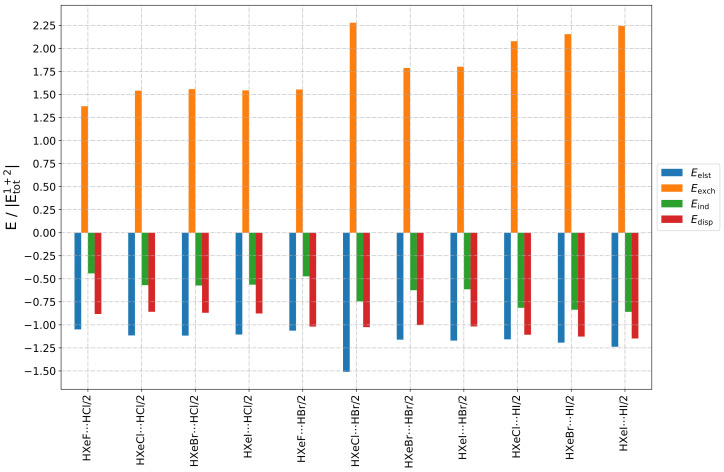
Bar graphs depicting the SAPT decomposition of the intermolecular interaction energy, using values relative to the total interaction energy, for HXeY⋯HX complexes assuming Type 2 structure. The graphs are grouped by HX molecule.

**Figure A6:**
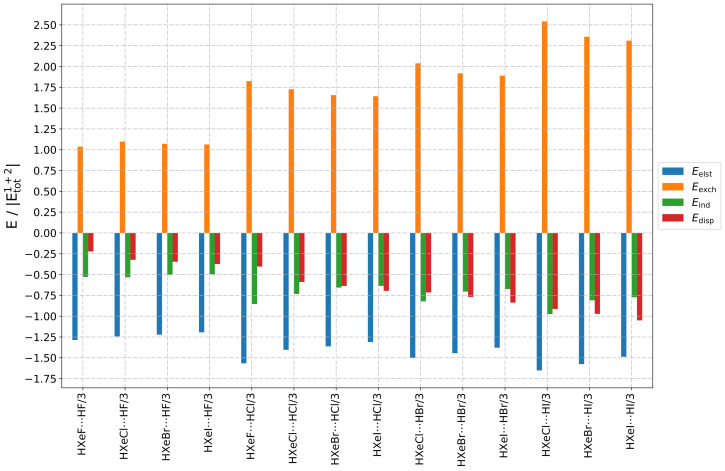
Bar graphs depicting the SAPT decomposition of the intermolecular interaction energy, using values relative to the total interaction energy, for HXeY⋯HX complexes assuming Type 3 structure. The graphs are grouped by HX molecule.

**Figure A7:**
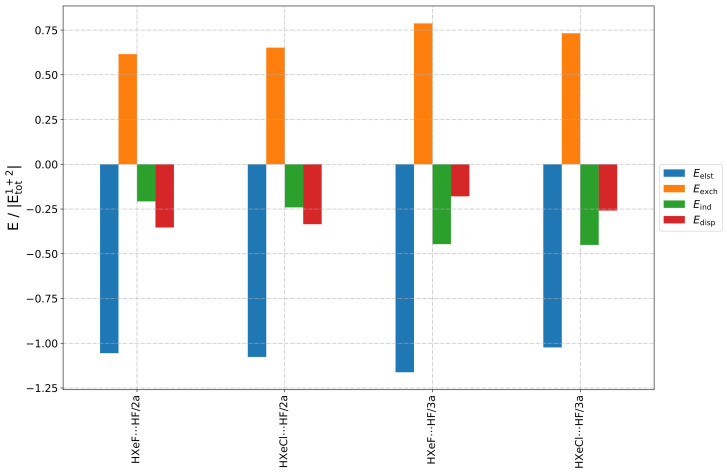
Bar graphs depicting the SAPT decomposition of the intermolecular interaction energy, using values relative to the total interaction energy, for HXeY⋯HX complexes assuming Type 2a and 3a structures.

**Figure A8:**
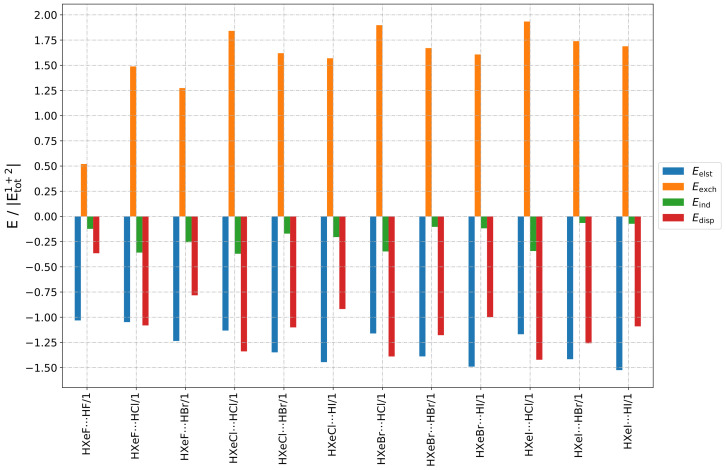
Bar graphs depicting the SAPT decomposition of the intermolecular interaction energy, using values relative to the total interaction energy, for HXeY⋯HX complexes assuming Type 1 structure. The graphs are grouped by HXeY molecule.

**Figure A9:**
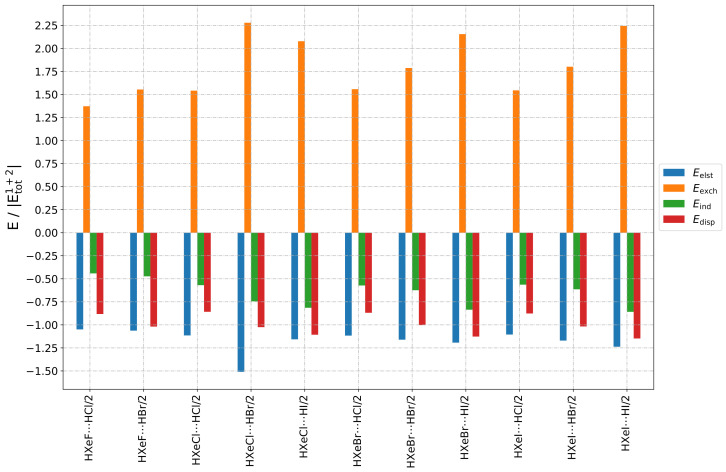
Bar graphs depicting the SAPT decomposition of the intermolecular interaction energy, using values relative to the total interaction energy, for HXeY⋯HX complexes assuming Type 2 structure. The graphs are grouped by HXeY molecule.

**Figure A10:**
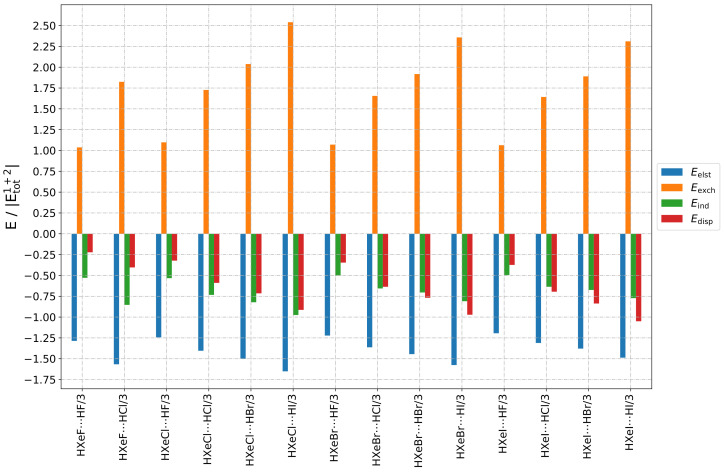
Bar graphs depicting the SAPT decomposition of the intermolecular interaction energy, using values relative to the total interaction energy, for HXeY⋯HX complexes assuming Type 3 structure. The graphs are grouped by HXeY molecule.

The results are presented as follows, in a group-wise manner according to the structure types indicated in Section 2.1. The numerical results of the total SAPT energy are presented in Table 1. Furthermore, three types of graphs are shown, to facilitate different types of comparisons. In Figure 2, Figure 3, Figure 4 and Figure 5, values for different energy terms for the complexes grouped by the HX molecule are presented. In Figure A1, Figure A2 and Figure A3, the same results are arranged by the HXeY molecule (the Type 2a and Type 3a results were not rearranged, due to their limited number). In the Appendix A, in Figure A4, Figure A5, Figure A6, Figure A7, Figure A8, Figure A9 and Figure A10, are the relative values of different components of the interaction energy. Accordingly, each individual term was divided by $E_{\mathrm{tot}}^{1+2}$, prior to plotting, the total SAPT interaction energy being represented by 1: this provided insight into the nature of the interaction, abstracting it from the magnitude of the interaction as a whole, which could be understood as a qualitative “fingerprint” of the interaction.

The SAPT contributions to the total interaction energy of the Group 1 structures are presented in Figure 2 and Figure A1. In this type of geometry, the HX molecule faces the halogen atom of HXeY, and the Xe–Y⋯X angle is acute (see Figure 1a). The main sources of stabilization are the electrostatic and dispersive terms, while induction plays a minor role. Dispersion was clearly the dominant second-order contribution to the interaction energy. For a given HX molecule, the individual contributions remained remarkably constant. For a given HXeY molecule in this structure type, the electrostatic, exchange, and dispersion terms increased significantly when changing the mass of HX, which produced the increasing total interaction energy. This was in line with our supermolecular results (see Section 2.2).

The relative contributions to the total interaction energy provided a qualitative picture of the interaction energy composition, i.e., they formed a fingerprint of the interaction type. The relative contributions are depicted in Figure A4 and Figure A10: one can see that the fingerprint remained generally the same for all these structures, independent of the composition.

The low induction contribution may come as a surprise, because the noble-gas molecules had large dipole moments; however, one may note that the dipole moments of both interacting molecules pointed towards similar directions, i.e., no large dipole moment induction should have been expected. Thus, there were no co-operative forces to induce additional partial charges.

This was in stark contrast to what was observed for energy partitioning for Type 3 structures. These structures generally had the lowest total interaction energy, which made them potentially the most stable. Their SAPT energies are presented in Figure 4 and Figure A3. For these geometries, the hydrogen atom from HX faced the halogen atom from HXeY, and the Xe–Y⋯H angle was acute (Figure 1d). These structures depicted a hydrogen bond HXeY⋯HX interaction; however, as they were strongly bent, a larger interaction between entire constituent molecules was also expected. This is evidenced by the bar graphs in Figure 4. One can see that the first-order energies provided little stabilizing effect, or were destabilizing, while the second-order induction and dispersion provided crucial attractive contributions to the total interaction. This shows that the Type 3 complexes were induction–dispersion-stabilised, whereas induction played the leading role in most of the structures. The role of dispersion increased with the atomic number of the halogen atom. In general terms, the magnitude of the induction and dispersion interactions was comparable for all Type 3 structures, especially for a given HXeY molecule, with the exception of those containing F. It can be concluded that the observed weakening of the total interaction, when going from HF to HI, was caused by the increasing electrostatic and exchange terms. The same trend could be observed for a fixed HX.

The relative contributions of the individual terms to the total interaction (see Figure A6) were quite similar for all Type 3 structures, but there was a slight variation in dispersion-to-induction ratio, as noted above: the ratio changed from ca. 0.5 to 1.33. Summarising these trends, it appears that the share of $E_{\mathrm{disp}}$, when compared to $E_{\mathrm{ind}}$, was highest for the HXeI⋯HI structure, and lowest for HXeF⋯HF. Additionally, for Type 3 structures, both the induction and dispersion interactions were the largest, compared to the other groups of the studied complexes.

In the same way as the small induction terms could be rationalized by the orientation of the interacting molecules for the Type 1 structures, the large induction interaction for the Type 3 structures could be explained by the dipole moments being oriented in an advantageous manner relative to one other, i.e., they were close to antiparallel orientation.

The SAPT energy contributions for the Type 2 structures are presented in Figure 3 and Figure A2: of all the considered types of structures, they had the highest total energy in general and, therefore, they were potentially the least stable complexes. The halogen atom of HX faced the hydrogen atom of HXeY, while the angle H-X⋯H was close to 90 degrees (see Figure 1b). The structures were stabilised by electrostatic, induction, and dispersion energies, with the contributions of all three being important; however, as the electrostatic term was again quenched by the exchange interaction, it was straightforward that the stabilisation of these complexes arose mostly from the second-order corrections, i.e., dispersion and induction. Unlike the Type 3 structures, the Type 2 structures were clearly dominated by the dispersion contribution. Both induction and dispersion fell in order from HF to HI for the structures with a given HXeY. Similarly, both energy contributions fell in order from HXeF to HXeI for the structures with a given HX; however, this fall was generally smoother. There was an exception in the trends for structures with HXeF, in which the relative contribution of induction (see Figure A9) and dispersion rose slightly in order from HF to HI. On the relative graphs, one can observe that the overall fingerprint of the interaction remained similar for all the Type 2 structures.

These energetic characteristics of the Type 1, 2, and 3 structures were in agreement with a previous report for HXeY⋯HX for X, Y = Cl and Br (see Table 4 in Ref. [14]); however, direct comparison is difficult, because of the different methodology employed: namely, the Morokuma analysis. The present results show that trends in energy decomposition apply to different compositions of the complexes, and depend mostly on the geometry.

The Type 2a structures were similar to the Type 2 structures: the HX halogen atom faced the hydrogen atom of HXeY, but with the difference that the structure was linear, i.e., the angle H–X⋯H was close to 180 degrees (see Figure 5). Only two structures of this kind were found to converge, and both these cases contained HF: for these structures, the relative share of electrostatic energy was much higher than the share of dispersion and induction energies. Compared to this, the structure with HXeCl had a lower total interaction energy, along with all other energy components. The destabilisation arose from greater exchange energy; moreover, the share of the induction energy compared to the dispersion energy was much higher for the structure with HXeCl (see Figure A7).

Analogically, the Type 3a structures were similar to the Type 3 structures, the only difference being that the Xe–Y⋯H angle was obtuse. Only two such structures, containing HF, were successfully converged. Again, the share of electrostatic energy was much higher than the share of dispersion and induction energies. Here, the structure with HXeCl had higher total energy, which made it potentially less stable. The induction and electrostatic energies were clearly higher in the structure with HXeF, whereas the dispersion energy was lower. The destabilisation coming from the exchange energy was also lower. The share of induction compared to dispersion was significantly lower for the structure containing the HXeCl molecule.

### 2.4. Vibrational Spectroscopy

In this section, the focus is on features of the computed anharmonic vibrational spectra that are particularly interesting from the experimental point of view, i.e., the predicted wavenumbers of the stretching vibrational mode of Xe–H, $\nu_{\mathrm{Xe}-H}$, and the stretching vibrational mode of H–X, $\nu_{H-X}$. The point of this discussion is to analyse the influence of the complexation on each property. The results of $\nu_{\mathrm{Xe}-H}$ are collected in Table 2, Table 3, Table 4, Table 5 and Table 6, and the wavenumbers for $\nu_{H-X}$ are collected in Table 7, Table 8, Table 9, Table 10 and Table 11. The analysis was based on experimental values regarding the complexation effect, from existing literature resources, and the effect accounted for by the computational MP2 and B3LYP methods. Where we were unable to obtain a numerical value of a frequency, or where the literature data on a particular species were not available, we marked these cases with a hash character (#) in the tables.

Especially, Xe–H vibrational frequencies for isolated HNgY molecules were overestimated drastically on the levels used here. It has been previously suggested that using more sophisticated correlation methods to compute the harmonic spectra (such as CCSD(T)), and to effectively correct that with lower-level computed anharmonicity (such as MP2), can result in a reasonably good correlation between computations and experimental matrix isolation findings [45,46]. The importance of the anharmonicity of the HXeY molecules was noted by Runeberg et al. [47] on HXeH, indicating that the multireference PES, when stretching the molecular bonds, was connected to the mismatch of vibrational fundamental modes between heavy ab initio calculations and experimental matrix isolation data; therefore, the abovementioned “CCSD(T) harmonic + MP2 anharmonic approach” could be considered a cost-effective way to predict IR features, compared to expensive and extensive molecular computations, in order to support and identify experimental vibrational features of systems involving noble-gas species, such as HXeY, studied here.

#### 2.4.1. Xe–H Stretching Mode

As seen in Table 2, Table 3, Table 4, Table 5 and Table 6, 32 of the 41 structures studied exhibited a blueshift of the Xe–H stretching mode: in accordance with previous studies [14,15,31,33,35,36], this shows that this behaviour was typical for the investigated compounds, even when the noble-gas molecule was not the proton donor. Two methodological points should be noted: firstly, the DFT/B3LYP/aug-cc-pVTZ(-PP) model seemed not to perform well, because it predicted redshifts for some of the structures, e.g., HXeCl⋯HCl/1, while the blueshifts seemed to be experimentally confirmed; secondly, although our monomer wavenumber values were overestimated by about 200 cm${}^{-1}$, compared to the reported experimental results, the blueshifted values were under a minor influence of anharmonicity. For example, Lignell et al. [14] studied the HXeY⋯HX complexes for Y, X = Cl and Br, and reported the experimental range observed for the blueshifted $\nu_{\mathrm{Xe}-H}$ for HXeCl⋯HCl to be 30.1–115.5 cm${}^{-1}$; their harmonic computational results for the same range were 10–117 cm${}^{-1}$, which agreed with our MP2 anharmonic results: 8–109 cm${}^{-1}$ (see Table 3). The results for HXeCl⋯HBr and HXeBr⋯HBr and HXeBr⋯HCl compared equally well (see Table 4).

The blueshifts were the largest for Type 3 structures, which can be traced back to their large interaction energies, i.e., stronger interaction and larger perturbation of electronic structures upon complexation, as discussed above. Their values ranged from 106 to 154 cm${}^{-1}$. The largest blueshifts were observed for complexes containing HF. The shifts for geometries of Types 1 and 2 of all compositions were positive, and ranged from a couple of units to 59 cm${}^{-1}$. The only exceptions to this blueshifting tendency were the complexes with HI, which—according to our anharmonic results—mostly exhibited a redshift.

The curious exception of complexes with HI is puzzling. We did not observe any drastic differences between this complex and the others, in terms of interaction energy components; however, the HXeCl⋯HI/3 complex was the least stable in the HXeCl⋯HX/3 group. HI also has the smallest dipole moment (see Table 12). From the HXeCl⋯HI complexes, the HXeCl⋯HI/2 structure had the lowest interaction energy and the largest redshift, of −365 cm${}^{-1}$. Zhu et al. [36] noted that no experimental evidence for the HXeCl⋯HI complex was found, but their calculations predicted a blueshift. Our results, however, suggest that the vibrational bands corresponding to complexes with HI should be sought for wavenumbers lower, compared to the isolated noble-gas molecule. Tsuge et al. [35] did indeed find computationally a redshifting H–Xe stretching mode, but only for the Type 2 structure of HXeI⋯HI, and for another dihydrogen-bonded structure not studied here. Perhaps in the case of HI depicting lower interaction energies, the same effect that causes the blueshift of $\nu_{\mathrm{XeH}}$ for other complexes does not occur. On the other hand, some part of the interaction mechanism may not been accounted for in our calculations: for example, a better description of relativistic effects—applying more sophisticated electron correlation methods or taking into account intramolecular BSSE for the HXeY molecule in the complex—could be considered [47]. This could also give insights into blue/redshift mismatch, in case there are more fundamental halogen-bond-type interactions in the interactive triangle structures of heavy atoms in Type 1 structures [48].

The results for complexes with HXeF provided two more redshifting cases: namely, the HXeF⋯HBr/2 and HXeF⋯HCl/3 structures; the first of these, however, had a redshift of −3 cm${}^{-1}$, which may have been a computational artefact. The latter complex redshifted substantially, but there are no experimental data to refer to. In general, we were not successful in finding all the structures for HXeF complexes, but for those reported in Table 2 the observed trends were similar to the other noble-gas molecules.

#### 2.4.2. Hydrogen Halide H–X Stretching Mode

Another interesting vibrational mode is that of the HX molecule. In Table 7, Table 8, Table 9, Table 10 and Table 11, one can see that all the $\nu_{H-X}$ modes were redshift, and that the magnitude of the complexation effect corresponded to the intermolecular interaction energy. Consequently, the HXeCl/Br/I Type 3 structures, in which HX was the proton donor, showed the largest redshifts—approximately −300 to −500 cm${}^{-1}$—while the Types 1 and 2 structures ranged from a few to approximately −40 cm${}^{-1}$. In the HXeF complexes, which were not synthesised, the computational redshift of HF reached −1000 cm${}^{-1}$.

This trend can be seen in each subset of the Type 1 structures, except for the HXeF molecule. For example, in the HXeCl⋯HCl/HBr/HI complexes, the redshift increased as the interaction energy increased. This trend was reversed for the Type 2 structures.

The obtained redshifted values for the HXeI complex were in agreement with previous experimental studies: for instance, the change registered by Zhu et al. for HXeI⋯HCl/3 (see Ref. [36], Table V) was −337 cm${}^{-1}$, and our anharmonic value was equal to −357 cm${}^{-1}$. The experimental shift for the Type 3 complex with HBr was −378 cm${}^{-1}$ [35], and our calculations provided the value of −363 cm${}^{-1}$. Our results could be used as a corroboration of the experimental assignments from the literature: for example, Tsuge et al. suggested another $\nu_{\mathrm{HBr}}$ band that was redshifted by −266 cm${}^{-1}$, but this value was quite far from the anharmonic results; similarly, the same authors observed a redshift of −167 cm${}^{-1}$ for HI in complexes with HXeI/3, but we predict a number twice as large—−347 cm${}^{-1}$.

The computed redshifts of $\nu_{H-F}$ for complexes with the HXeF molecule were very large: −863 cm${}^{-1}$ for HXeF⋯HF/3, and even −1001 cm${}^{-1}$ in the case of HXeF⋯HCl/3. The former value compared reasonably well to a previous study by Yen et al. [49]. These authors obtained a redshift of −668 cm${}^{-1}$ for HXeF⋯HF/3. Jankowska and Sadlej [38] reported a value of −728 cm${}^{-1}$.

## 3. Computational Methods

Geometry optimisation was performed, using numerous starting molecular orientations of the HX and HXeY molecules, with the aim of finding as many local energy minima as possible. Optimization of the complex structures was conducted, employing the Boys–Bernardi full counterpoise method by Dannenberg [50,51]. Vibrational analysis was performed on the converged structures, to exclude transition states. The anharmonic vibrational frequencies were obtained using the vibrational second-order perturbation theory (VPT2) [52,53], as implemented in the Gaussian 09 [54] program. These procedures were performed using density functional theory with the B3LYP functional.

Further calculations were intended to determine the supermolecular intermolecular interaction energy and the physical nature of the interactions, by means of symmetry-adapted perturbation theory (SAPT) [41], as implemented in the MOLPRO software suite [55].

Preceding the computations of the intermolecular interaction energies, additional geometry optimisation was applied to previously found structures, this time using the second-order Møller–Plesset perturbation theory (MP2) in the Gaussian programme suite. Not all structures previously found were successfully optimised, including most of the structures with linear geometry (later denoted as Type 2a structures; see Section 2.1). For some of the uncertain structures, additional calculations were performed, using “very tight” convergence criteria. The natures of all the minima were established by vibrational analysis, and anharmonic frequencies were also calculated. Only the anharmonic vibrational frequencies are discussed herein, and are presented in the tables.

For hydrogen, fluorine, and chlorine, the Dunning-type singly augmented aug-cc-pVTZ basis set was employed [56,57,58,59]; meanwhile, for the heavier elements—i.e., bromine, iodine, and xenon—the aug-cc-pVTZ-PP basis set [60,61] was employed, which took into account the scalar relativistic effect, employing the effective core potentials (ECP). The basis sets were obtained from the Basis Set Exchange (BSE) library [62].

The supramolecular interaction energy was estimated on different levels of theory by the standard approach, i.e., subtracting the total energies of the constituent species from the total energy of the complex:

(1) $$E_{\mathrm{int}}^{\mathrm{method}}=E_{A+B}^{\mathrm{method}}-E_{A}^{\mathrm{method}}-E_{B}^{\mathrm{method}},$$

where $E_{\mathrm{int}}$ represents the interaction energy, $E_{A+B}$ represents the energy of the system consisting of both molecules interacting with each other, $E_{A}$ and $E_{B}$ represent the energies of molecules A and B, and the superscript denotes that they were all obtained by the same method. $E_{A}$ and $E_{B}$ were calculated in the full basis set of the dimer, by zeroing the atomic charge of the partner molecule. By this approach, we took into account the basis set superposition error (BSSE) [50]. We expected the energy correction coming from BSSE procedure to be important, because of the size of the studied systems, e.g., the correction to the total energy of HX coming from the inclusion of basis set functions from HXeY molecules containing a heavy atom was expected to be substantial: this was indeed confirmed in the calculation, e.g., the BSSE correction for the most stable HXeCl complex with HCl was ca. 5 kJ/mol. The employed methods included the Hartree–Fock method (HF), the second- and fourth-order Møller–Plesset perturbation theories (MP2 and MP4), coupled cluster singles and doubles (CCSD), and coupled cluster singles doubles with perturbative triples (CCSD(T)). Because one of our goals was to juxtapose the supramolecular energy values and the SAPT values, zero-point energy (ZPE) corrections were not employed, nor the corrections for geometry deformation upon complexation. However, as an illustration, for the most stable of the complexes of HXeCl with HCl, the deformation correction to the complexation energy, $\Delta E_{\mathrm{def}}$, was found to be of order ca. 2 kJ/mol, and the ZPE correction was ca. 5 kJ/mol.

At the theory level employed, the SAPT interaction energy had the following components:

(2) $$E_{\mathrm{int}}^{\mathrm{SAPT}0}=E_{\mathrm{elst}}^{\left(10\right)}+E_{\mathrm{exch}}^{\left(10\right)}+E_{\mathrm{ind},r}^{\left(20\right)}+E_{\mathrm{exch}-\mathrm{ind},r}^{\left(20\right)}+E_{\mathrm{disp}}^{\left(20\right)}+E_{\mathrm{exch}-\mathrm{disp}}^{\left(20\right)}+\delta E_{\mathrm{int},\mathrm{resp}}^{\mathrm{HF}},$$

where $E_{\mathrm{elst}}^{\left(10\right)}$ was electrostatic energy, $E_{\mathrm{exch}}^{\left(10\right)}$ was exchange energy, $E_{\mathrm{ind},r}^{\left(20\right)}$ was induction energy, $E_{\mathrm{exch}-\mathrm{ind},r}^{\left(20\right)}$ was coupling between exchange and induction energy, $E_{\mathrm{disp}}^{\left(20\right)}$ was dispersion energy, $E_{\mathrm{exch}-\mathrm{disp}}^{\left(20\right)}$ was coupling between exchange and dispersion energy, and $\delta E_{\mathrm{int},\mathrm{resp}}^{\mathrm{HF}}$ was assumed to include mainly the third-order induction term, and was obtained by subtracting the sum of all the previous terms from the pure Hartree–Fock energy. The superscript indicates the order of terms in the perturbative expansion. Qualitatively, the electrostatic term describes the interaction of the unperturbed multipoles of each interacting molecule. The induction terms describe the interaction of the unperturbed multipoles of one molecule with the induced multipoles of the other, while the dispersive terms correspond to the interaction of the induced multipoles of both molecules. The exchange terms arise from the application of the antisymmetrization operator: they are a purely quantum effect, and they are positive.

The above terms were arranged as follows for our analysis:

$$E_{\mathrm{elst}}=E_{\mathrm{elst}}^{\left(10\right)}$$

$$E_{\mathrm{exch}}=E_{\mathrm{exch}}^{\left(10\right)}$$

$$E_{\mathrm{ind}}=E_{\mathrm{ind},r}^{\left(20\right)}+E_{\mathrm{exch}-\mathrm{ind},r}^{\left(20\right)}+\delta E_{\mathrm{int},\mathrm{resp}}^{\mathrm{HF}}$$

$$E_{\mathrm{disp}}=E_{\mathrm{disp}}^{\left(20\right)}+E_{\mathrm{exch}-\mathrm{disp}}^{\left(20\right)}$$

$$E_{\mathrm{tot}}^{\mathrm{int}}=E_{\mathrm{tot}}^{1+2}=E_{\mathrm{elst}}+E_{\mathrm{exch}}+E_{\mathrm{ind}}+E_{\mathrm{disp}}$$

and were plotted in the form of bar graphs.

All data analyses and visualization were performed using iPython and Jupyter Notebooks [63,64], Matplotlib [65], and Pandas [66,67] packages.

## 4. Conclusions

A structural search, an analysis of the intermolecular interaction energy, and an anharmonic Xe–H and H–X stretching vibrational mode analysis for the total of 41 different complexes of the formula HXeY⋯HX were performed. Several previously unknown structures of the complexes are reported. Based on the computational results, different complex structures exhibited characteristic patterns, in terms of electrostatic/induction/dispersion contributions to the total intermolecular interaction energy, as follows:

1. Type 1 structures were mostly stabilized by dispersion interaction;
2. Type 2 structures had large contributions from all three types of interaction; however, as the first-order energy was repulsive, the second-order terms were the most important ones for stabilization of the complex structures;
3. Type 3 structures were induction–dispersion-stabilized, and induction played a major role in their complex formation;
4. Type 2a and 3a structures were outliers to these patterns: the electrostatic energy had the largest values of all. From the second-order terms, induction was more important for Type 3a structures, while dispersion was slightly more important in the case of Type 2a structures.

Almost all of the studied structures exhibited a blueshift of the Xe–H stretching vibrational mode, except for most of the complexes of HI and the complexes of HXeF. The magnitude of the complexation effect roughly correlated to the magnitude of the interaction, and the induction-stabilized complexes showed the largest shifts. The stretching vibrational mode of H–X was found to redshift, and this effect was greatest for the Type 3 structures.

## Figures and Tables

**Table 1:** The supermolecular interaction energy values obtained by HF, MP2, MP4, CCSD, and CCSD(T) methods, and the total SAPT interaction energy of all the examined structures, given in kJ·mol${}^{-1}$.

Complex/Structure Type	SAPT	HF	MP2	MP4	CCSD	CCSD(T)
HXeF┄ HF/1	−11.04	−7.48	−7.57	−6.97	−7.54	−7.39
HXeF┄ HCl/1	−8.03	1.19	−3.69	−2.99	−2.41	−2.92
HXeF┄ HBr/1	−17.63	−3.29	−9.37	−8.45	−7.73	−8.32
HXeCl┄ HCl/1	−7.54	2.95	−4.61	−3.14	−2.07	−2.80
HXeCl┄ HBr/1	−18.00	1.45	−9.07	−6.97	−5.45	−6.41
HXeCl┄ HI/1	−32.62	−1.63	−14.18	−11.36	−9.42	−10.58
HXeBr┄ HCl/1	−7.59	3.33	−4.75	−3.16	−2.00	−2.76
HXeBr┄ HBr/1	−19.03	2.37	−8.93	−6.65	−4.99	−6.01
HXeBr┄ HI/1	−36.00	−0.04	−13.72	−10.67	−8.53	−9.77
HXeI┄ HCl/1	−7.37	3.52	−4.74	−3.05	−1.83	−2.61
HXeI┄ HBr/1	−19.28	3.21	−8.41	−6.00	−4.23	−5.28
HXeI┄ HI/1	−37.75	1.63	−12.65	−9.41	−7.11	−8.40
HXeF┄ HCl/2	−5.48	−0.53	−3.79	−2.58	−2.40	−2.66
HXeF┄ HBr/2	−6.32	0.58	−3.88	−2.57	−2.15	−2.54
HXeCl┄ HCl/2	−6.25	−1.13	−5.30	−3.81	−3.17	−3.47
HXeCl┄ HBr/2	−6.09	0.19	−5.69	−3.93	−2.96	−3.44
HXeCl┄ HI/2	−9.25	1.57	−6.28	−4.13	−2.71	−3.43
HXeBr┄ HCl/2	−6.08	−1.05	−4.97	−3.42	−2.76	−3.02
HXeBr┄ HBr/2	−8.22	0.33	−5.43	−3.55	−2.52	−2.97
HXeBr┄ HI/2	−10.16	1.85	−6.17	−3.78	−2.23	−2.93
HXeI┄ HCl/2	−5.82	−1.00	−4.39	−2.75	−2.12	−2.30
HXeI┄ HBr/2	−8.54	0.45	−4.93	−2.87	−1.80	−2.16
HXeI┄ HI/2	−11.65	2.17	−5.89	−3.10	−1.38	−2.00
HXeF┄ HF/3	−67.09	−65.91	−62.99	−59.53	−63.05	−61.96
HXeF┄ HCl/3	−38.80	−41.08	−51.87	−46.10	−45.48	−46.36
HXeCl┄ HF/3	−43.05	−35.43	−41.10	−39.13	−38.59	−38.83
HXeCl┄ HCl/3	−26.02	−16.49	−32.85	−28.22	−25.54	−27.18
HXeCl┄ HBr/3	−24.93	−10.66	−29.73	−24.64	−21.26	−23.33
HXeCl┄ HI/3	−20.15	−2.81	−24.24	−18.71	−14.53	−17.10
HXeBr┄ HF/3	−39.21	−29.81	−35.43	−33.54	−32.92	−33.17
HXeBr┄ HCl/3	−26.28	−12.97	−28.92	−24.53	−21.89	−23.43
HXeBr┄ HBr/3	−27.66	−7.84	−26.57	−21.70	−18.40	−20.35
HXeBr┄ HI/3	−25.67	−0.95	−22.23	−16.88	−12.78	−15.22
HXeI┄ HF/3	−33.30	−23.92	−28.90	−27.12	−26.46	−26.66
HXeI┄ HCl/3	−22.91	−9.18	−24.01	−19.95	−17.42	−18.82
HXeI┄ HBr/3	−25.56	−4.72	−22.48	−17.92	−14.71	−16.52
HXeI┄ HI/3	−25.36	1.23	−19.30	−14.23	−10.23	−12.51
HXeF┄ HF/2a	−10.31	−7.31	−6.91	−5.90	−6.72	−6.45
HXeCl┄ HF/2a	−12.23	−9.64	−9.22	−8.21	−8.60	−8.23
HXeF┄ HF/3a	−63.67	−63.86	−58.51	−55.56	−59.25	−57.86
HXeCl┄ HF/3a	−31.87	−27.68	−29.32	−27.98	−28.04	−27.83

**Table 2:** The anharmonic $\nu_{\mathrm{Xe}-H}$ stretching vibration frequencies in the studied complexes in different structures of HXeF complexes: Δν denotes the change in value compared to a non-interacting monomer. Values are given in ${\mathrm{cm}}^{-1}$. There is no experimental value of $\nu_{\mathrm{Xe}-H}$ for the HXeF molecule. The character # indicates there is no data available.

Type	HX	ν(MP2)	Δν(MP2)	ν(B3LYP)	Δν(B3LYP)
1	HF	2037	29	#	#
	HCl	2010	2	1833	−81
	HBr	2022	14	1949	35
	HI	#	#	#	#
2	HF	#	#	#	#
	HCl	2020	12	1922	8
	HBr	2005	−3	1902	−12
	HI	#	#	#	#
3	HF	2127	119	1945	31
	HCl	1659	−349	1942	28
	HBr	#	#	#	#
	HI	#	#	#	#

**Table 3:** The anharmonic $\nu_{\mathrm{Xe}-H}$ stretching vibration frequencies in the studied complexes in different structures of HXeCl complexes: Δν denotes the change in value compared to a non-interacting monomer that equals 1648 cm${}^{-1}$ (experiment; see Refs. [7,20]) and 1825 cm${}^{-1}$ (our anharmonic calculations). Values are given in ${\mathrm{cm}}^{-1}$. The character # indicates there is no data available.

Type	HX	Δν(exp)	ν(MP2)	Δν(MP2)	ν(B3LYP)	Δν(B3LYP)
1	HF	#	#	#	#	#
	HCl	30–50	1833	8	1730	−19
	HBr	#	1847	22	1759	10
	HI	#	1611	−214	1774	25
2	HF	#	#	#	#	#
	HCl	30–50	1857	32	1779	30
	HBr	#	1828	3	1720	−29
	HI	#	1460	−365	1603	−146
3	HF	#	1957	132	1831	82
	HCl	80–115	1934	109	1750	1
	HBr	#	1931	106	1858	109
	HI	#	1659	−166	1733	−16

**Table 4:** The anharmonic $\nu_{\mathrm{Xe}-H}$ stretching vibration frequencies in the studied complexes in different structures of HXeBr complexes: Δν denotes the change in value compared to a non-interacting monomer that equals 1504 cm${}^{-1}$ (experiment; see Refs. [7,20]) and 1710 cm${}^{-1}$ (our anharmonic calculations). Values are given in ${\mathrm{cm}}^{-1}$. The character # indicates there is no data available.

Type	HX	Δν(exp)	ν(MP2)	Δν(MP2)	ν(B3LYP)	Δν(B3LYP)
1	HF	#	#	#	#	#
	HCl	#	1719	9	1668	101
	HBr	24–39	1734	24	1679	112
	HI	#	1755	45	1693	126
2	HF	#	#	#	#	#
	HCl	#	1755	45	#	#
	HBr	24–39	1729	19	1542	−25
	HI	#	1660	−50	1388	−179
3	HF	#	1852	142	1709	142
	HCl	80–120	1827	117	1668	101
	HBr	73–145	1826	116	1732	165
	HI	#	1774	64	1730	163

**Table 5:** The anharmonic $\nu_{\mathrm{Xe}-H}$ stretching vibration frequencies in the studied complexes in different structures of HXeI complexes: Δν denotes the change in value compared to a non-interacting monomer that equals 1193 cm${}^{-1}$ (experiment; see Refs. [7,36]) and 1529 cm${}^{-1}$ (our anharmonic calculations). Values are given in ${\mathrm{cm}}^{-1}$. The character # indicates there is no data available.

Type	HX	Δν(exp)	ν(MP2)	Δν(MP2)	ν(B3LYP)	Δν(B3LYP)
1	HF		#	#	#	#
	HCl	94–155	1537	8	1361	−64
	HBr		1554	25	1383	−42
	HI		1279	−250	1391	−34
2	HF		#	#	#	#
	HCl	94–155	1588	59	#	#
	HBr		1571	42	1313	−112
	HI		1044	−485	1190	−235
3	HF		1683	154	1561	136
	HCl	94–155	1651	122	1533	108
	HBr		1650	121	1533	108
	HI		1330	−199	1506	81

**Table 6:** The anharmonic $\nu_{\mathrm{Xe}-H}$ stretching vibration frequencies in the studied complexes in different structures of Types 2a and 3a: Δν denotes the change in value compared to a non-interacting monomer. Values are given in ${\mathrm{cm}}^{-1}$. The character # indicates there is no data available.

Type	Structure	ν(MP2)	Δν(MP2)	ν(B3LYP)	Δν(B3LYP)
2a	HXeF⋯FH	2024	16	#	#
	HXeCl⋯FH	1881	56	#	#
3a	HXeF⋯FH	2126	118	#	#
	HXeCl⋯FH	1922	97	#	#

**Table 7:** The anharmonic $\nu_{X-H}$ stretching vibration frequencies in the studied complexes in different structures of HXeF complexes: Δν denotes the change in value compared to a non-interacting monomer. Values are given in ${\mathrm{cm}}^{-1}$. The character # indicates there is no data available.

Type	HX	ν(MP2)	Δν(MP2)	ν(B3LYP)	Δν(B3LYP)
1	HF	3923	−29	#	#
	HCl	2932	−11	2808	−52
	HBr	2641	−23	2568	40
	HI	#	#	#	#
2	HF	#	#	#	#
	HCl	2931	−12	2855	−5
	HBr	2653	−11	2523	−5
	HI	#	#	#	#
3	HF	3089	−863	3019	−882
	HCl	1942	−1001	1862	−998
	HBr	#	#	#	#
	HI	#	#	#	#

**Table 8:** The anharmonic $\nu_{X-H}$ stretching vibration frequencies in the studied complexes in different structures of HXeCl complexes: Δν denotes the change in value compared to a non-interacting monomer. Values are given in ${\mathrm{cm}}^{-1}$. The character # indicates there is no data available.

Type	HX	ν(MP2)	Δν(MP2)	ν(B3LYP)	Δν(B3LYP)
1	HF	#	#	#	#
	HCl	2928	−15	2861	1
	HBr	2637	−27	2459	−69
	HI	2308	−38	2101	−105
2	HF	#	#	#	#
	HCl	#	#	2847	−13
	HBr	2651	−13	2524	−4
	HI	2337	−9	2209	3
3	HF	3370	−582	3302	−599
	HCl	#	#	2279	−581
	HBr	2174	−490	1942	−586
	HI	2024	−322	1776	−430

**Table 9:** The anharmonic $\nu_{X-H}$ stretching vibration frequencies in the studied complexes in different structures of HXeBr complexes: Δν denotes the change in value compared to a non-interacting monomer. Values are given in ${\mathrm{cm}}^{-1}$. The character # indicates there is no data available.

Type	HX	ν(MP2)	Δν(MP2)	ν(B3LYP)	Δν(B3LYP)
1	HF	#	#	#	#
	HCl	2928	−15	2862	2
	HBr	2634	−30	2403	−125
	HI	2305	−41	2087	−119
2	HF	#	#	#	#
	HCl	2927	−16	#	#
	HBr	2651	−13	2524	−4
	HI	2336	−10	2205	−1
3	HF	3475	−477	3376	−525
	HCl	2522	−421	2363	−497
	HBr	2244	−420	1924	−604
	HI	1961	−385	1758	−448

**Table 10:** The anharmonic $\nu_{X-H}$ stretching vibration frequencies in the studied complexes in different structures of HXeI complexes: Δν denotes the change in value compared to a non-interacting monomer. Values are given in ${\mathrm{cm}}^{-1}$. The character # indicates there is no data available.

Type	HX	ν(MP2)	Δν(MP2)	ν(B3LYP)	Δν(B3LYP)
1	HF	#	#	#	#
	HCl	2926	−17	2868	8
	HBr	2633	−31	2367	−161
	HI	2303	−43	2204	−2
2	HF	#	#	#	#
	HCl	2928	−15	#	#
	HBr	2652	−12	2525	−3
	HI	2336	−10	2197	−9
3	HF	#	#	3443	−458
	HCl	2587	−356	2343	−517
	HBr	2301	−363	2073	−455
	HI	1999	−347	1749	−457

**Table 11:** The anharmonic $\nu_{X-H}$ stretching vibration frequencies in the studied complexes in different structures of Types 2a and 3a: Δν denotes the change in value compared to a non-interacting monomer. Values are given in ${\mathrm{cm}}^{-1}$. The character # indicates there is no data available.

Type	Structure	ν(MP2)	Δν(MP2)	ν(B3LYP)	Δν(B3LYP)
2a	HXeFFH	3931	−21	#	#
	HXeClFH	3925	−27	#	#
3a	HXeFFH	3301	−651	#	#
	HXeClFH	3692	−260	#	#

**Table 12:** Dipole moment (in Debyes) of the HX and HXeY molecules.

Dipole Moments	HX	HXeY
H_2_O	1.85	1.85
F	1.82	2.05
Cl	1.08	6.42
Br	0.82	6.45
I	0.44	6.1

## Data Availability

Data available upon request.

## Abbreviations

The following abbreviations are used in this manuscript:

HF	Hartree–Fock
ZPE	Zero-Point Energy
SAPT	Symmetry-Adapted Perturbation Theory
MP2	Second-Order Møller–Plesset Theory
MP4	Fourth-Order Møller–Plesset Theory
CCSD	Coupled Cluster Singles and Doubles
CCSD(T)	Coupled Cluster Singles and Doubles with perturbative triples
DFT	Density Functional Theory
B3LYP	Becke, 3-parameter, Lee–Yang–Parr functional for DFT
BSE	Basis Set Exchange library
